# Improving antiretroviral therapy adherence in resource‐limited settings at scale: a discussion of interventions and recommendations

**DOI:** 10.7448/IAS.20.1.21371

**Published:** 2017-03-22

**Authors:** Jessica E. Haberer, Lora Sabin, K. Rivet Amico, Catherine Orrell, Omar Galárraga, Alexander C. Tsai, Rachel C. Vreeman, Ira Wilson, Nadia A. Sam‐Agudu, Terrence F. Blaschke, Bernard Vrijens, Claude A. Mellins, Robert H. Remien, Sheri D. Weiser, Elizabeth Lowenthal, Michael J. Stirratt, Papa Salif Sow, Bruce Thomas, Nathan Ford, Edward Mills, Richard Lester, Jean B. Nachega, Bosco Mwebesa Bwana, Fred Ssewamala, Lawrence Mbuagbaw, Paula Munderi, Elvin Geng, David R. Bangsberg

**Affiliations:** ^1^ Massachusetts General Hospital Global Health, Boston, MA, USA; ^2^ Department of Medicine, Harvard Medical School, Boston, MA, USA; ^3^ Department of Global Health, Center for Global Health and Department, Boston University School of Public Health, Boston, MA, USA; ^4^ Department of Health Behavior and Health Education, University of Michigan School of Public Health, Ann Arbor, MI, USA; ^5^ Desmond Tutu HIV Centre, Institute of Infectious Disease and Molecular Medicine, and Department of Medicine, University of Cape Town, Cape Town, South Africa; ^6^ Department of Health Services, Policy and Practice, Brown University School of Public Health, Providence, RI, USA; ^7^ Chester M. Pierce, MD Division of Global Psychiatry, Massachusetts General Hospital, Boston, MA, USA; ^8^ Department of Pediatrics, Indiana University School of Medicine, Indianapolis, IN, USA; ^9^ Academic Model Providing Access to Healthcare (AMPATH), Eldoret, Kenya; ^10^ Clinical Department, Institute of Human Virology Nigeria, Abuja, Nigeria; ^11^ Institute of Human Virology and Department of Pediatrics, University of Maryland School of Medicine, Baltimore, MD, USA; ^12^ Department of Medicine and Clinical Pharmacology, Stanford University School of Medicine, Stanford, CA, USA; ^13^ Department of Biostatistics, University of Liège, Liège, Wallonia, Belgium; ^14^ WestRock Healthcare, Sion, Switzerland; ^15^ HIV Center for Clinical and Behavioral Studies, NYSPI and Department of Psychiatry, Columbia; University, New York, NY, USA; ^16^ Division of HIV, ID and Global Medicine, Department of Medicine, University of California, San Francisco, CA, USA; ^17^ Departments of Pediatrics and Epidemiology, University of Pennsylvania, Perelman School of Medicine, Philadelphia, PA, USA; ^18^ Division of AIDS Research, National Institute of Mental Health, Bethesda, MD, USA; ^19^ Bill and Melinda Gates Foundation, Seattle, WA, USA; ^20^ Department of Infectious diseases, University of Dakar, Dakar, Sénégal; ^21^ The Arcady Group, LLC, Richmond, VA, USA; ^22^ Department of HIV/AIDS, World Health Organization, Geneva, Switzerland; ^23^ Faculty of Health Sciences, University of Ottawa, Ottawa, Ontario, Canada; ^24^ Division of Infectious Diseases, Department of Medicine, University of British Columbia; ^25^ Department of Epidemiology, University of Pittsburgh Graduate School of Public Health, Pittsburgh, PA, USA; ^26^ Department of Medicine, Mbarara University of Science and Technology, Mbarara, Uganda; ^27^ Columbia University School of Social Work & School of International and Public Affairs, New York, NY, USA; ^28^ Department of Health Research Methods, Evidence and Impact, McMaster University, Hamilton, Ontario, Canada; ^29^ HIV Care Research Program, Medical Research Council, Uganda Virus Research Institute, Entebbe, Uganda; ^30^ Division of HIV, Infectious Disease and Global Medicine, San Francisco General Hospital, Department of Medicine, University of California, San Francisco, CA, USA; ^31^ Oregon Health & Sciences University‐Portland State University School of Public Health, Portland, OR, USA

**Keywords:** HIV, antiretroviral therapy adherence, interventions, resource‐limited settings

## Abstract

**Introduction**: Successful population‐level antiretroviral therapy (ART) adherence will be necessary to realize both the clinical and prevention benefits of antiretroviral scale‐up and, ultimately, the end of AIDS. Although many people living with HIV are adhering well, others struggle and most are likely to experience challenges in adherence that may threaten virologic suppression at some point during lifelong therapy. Despite the importance of ART adherence, supportive interventions have generally not been implemented at scale. The objective of this review is to summarize the recommendations of clinical, research, and public health experts for scalable ART adherence interventions in resource‐limited settings.

**Methods**: In July 2015, the Bill and Melinda Gates Foundation convened a meeting to discuss the most promising ART adherence interventions for use at scale in resource‐limited settings. This article summarizes that discussion with recent updates. It is not a systematic review, but rather provides practical considerations for programme implementation based on evidence from individual studies, systematic reviews, meta‐analyses, and the World Health Organization Consolidated Guidelines for HIV, which include evidence from randomized controlled trials in low‐ and middle‐income countries. Interventions are categorized broadly as education and counselling; information and communication technology‐enhanced solutions; healthcare delivery restructuring; and economic incentives and social protection interventions. Each category is discussed, including descriptions of interventions, current evidence for effectiveness, and what appears promising for the near future. Approaches to intervention implementation and impact assessment are then described.

**Results and discussion**: The evidence base is promising for currently available, effective, and scalable ART adherence interventions for resource‐limited settings. Numerous interventions build on existing health care infrastructure and leverage available resources. Those most widely studied and implemented to date involve peer counselling, adherence clubs, and short message service (SMS). Many additional interventions could have an important impact on ART adherence with further development, including standardized counselling through multi‐media technology, electronic dose monitoring, decentralized and differentiated models of care, and livelihood interventions. Optimal targeting and tailoring of interventions will require improved adherence measurement.

**Conclusions**: The opportunity exists today to address and resolve many of the challenges to effective ART adherence, so that they do not limit the potential of ART to help bring about the end of AIDS.

## Introduction

Antiretroviral therapy (ART) has transformed HIV infection from a terminal disease into a manageable chronic illness [1]. ART can also reduce viral load (i.e. HIV RNA levels) and the risk of secondary transmission, thus defining a new HIV prevention paradigm in which successful treatment scale up could help realize an end to AIDS [2,3]. The vast majority of the 35 million people living with HIV (PLWH) are in resource‐limited settings (RLS), of whom an estimated 17 million were receiving ART in 2016 (up from 1.3 million people in 2006) [4]. Targets aim to increase the number on treatment to 20 million people by 2020 [4]. While this ART expansion represents one of the greatest public health achievements of our time, much remains to be done. Shifts in ART initiation guidelines, including “test and start” and Option B+, in which all HIV‐positive pregnant women start ART, highlight potential challenges for ART adherence and retention among ever‐growing numbers of PLWH [5,6].

The level of adherence required to achieve improved immune function and viral suppression varies, depending primarily on ART regimen and prior duration of viral suppression [7]; however, consistent dosing without sustained gaps is critical for realizing both the individual and public health benefits of ART [8–10]. ART adherence therefore plays a key role in achieving the second and third “90s” of the UNAIDS 90‐90‐90 campaign to end the AIDS epidemic (i.e. 90% on treatment and 90% virally suppressed) [11]. As used here, “adherence” encompasses (1) medication initiation, defined as taking the first dose – not simply receipt of a prescription; (2) dose‐taking execution (i.e. taking doses as prescribed) throughout treatment; and (3) treatment persistence, meaning continuing therapy without prolonged gaps (e.g. typically several weeks or longer) that signal a permanent or temporary termination of therapy [12]. Dose‐taking execution and persistence both require retention in care to facilitate timely prescription refills.

As ART access expands globally, medication adherence remains challenging. Factors associated with achieving and maintaining high ART adherence can be grouped according to the individual (e.g. knowledge, resources, mental health); interpersonal/network relationships (e.g. social support, stigma); the community (e.g. socio‐cultural norms); health system factors (e.g. service provision) and structural issues (e.g. access to services) [13]. Levels of medication adherence are difficult to assess given variability across available measures (discussed below); however, concern for incomplete adherence is high [14] and estimates of non‐adherence range widely from 2% to 70% [15]. According to a 2010 systematic review, one‐third of adults taking ART in sub‐Saharan Africa lose viral suppression at 2 years [16]. While some estimates of viral failure are lower than one‐third [17], others are higher for several reasons. First, adherence measured through research studies may be higher than that found in routine care, and most published studies only report on individuals actually retained in care. Second, current World Health Organization (WHO) guidelines promote ART initiation at high CD4 counts, when individuals may be asymptomatic [18] – studies focused on such patients indicate mixed results [5,19]. Additionally, the impact of new regimens (e.g. integrase inhibitors) and formulations in development (e.g. injectable long‐acting ART) on adherence is still unknown. Even with favorable circumstances, however, most PLWH are likely to experience challenges in adherence that threaten virologic suppression at some point during lifelong therapy.

This article discusses ART adherence interventions, specifically defined as strategies to improve adherence and/or relevant disease markers (i.e. HIV RNA, CD4 counts), and their potential for adaption and adoption across RLS. Interventions are considered broadly; however, some populations facing unique challenges (e.g. youth, pregnant/post‐partum women) are highlighted. Because the global scale up of ART to meet WHO targets of near universal coverage [18] will stress existing healthcare delivery systems, effective interventions are presented in the context of scalability, which is defined as (1) acceptance by PLWH and providers, (2) consistency with the supply chain of necessary resources, and (3) affordability from the perspective of the healthcare system providing ART.

## Methods

In July 2015, the Bill and Melinda Gates Foundation convened a meeting of clinical, research, and public health experts to discuss promising ART adherence interventions for use at scale in RLS. Specifically, experts were asked for interventions that could be implemented for thousands of patients or more. This article summarizes that discussion, with recent updates. Meeting participants included experts with experience with diverse types of ART interventions – targeting individual, social, community, health system, and other structural factors – as well as with multiple settings and key populations affected by HIV (e.g. youth, pregnant/post‐partum women, substance users, men who have sex with men, sex workers). The goal of this article is not to present a systematic review, but rather to summarize considerations for programme implementation based on evidence from individual studies, systematic reviews, meta‐analyses, and the WHO Consolidated Guidelines for HIV, including evidence from randomized controlled trials (RCTs), in RLS.

## Results

### Overview

Table 1 summarizes and provides examples of the interventions discussed at the meeting. These interventions can be categorized broadly as education and counselling; information and communication technology (ICT)‐enhanced solutions; healthcare delivery restructuring; and economic incentives and social protection interventions. Some interventions cut across multiple categories, reflecting combination approaches; these are organized by their dominant characteristic. Each category is discussed below, including descriptions of interventions, current evidence for effectiveness, and what appears promising for the near future. Approaches to intervention implementation and impact assessment are then described.

**Table 1:** Examples of ART adherence interventions with an impact on adherence and/or relevant disease markers for resource‐limited settings as discussed at the meeting and in the article*.

		Categories of intervention			
Interventions	Reference citations	Education and counselling	ICT‐enhanced solutions	Healthcare delivery restructuring	Economic incentives and social protection interventions
Cognitive behavioral therapy	[20–22]	X			
Motivational interviewing	[23]	X			
Treatment supporters/assigned community health workers	[24,25]	X		X	
Patient adherence clubs	[26]	X		X	
Peer supporter	[21,27–29]	X			
Peer‐delivered directly observed therapy	[30]	X			
Multimedia‐based adherence counselling	[31]	X	X		
SMS reminder messages	[32–34]		X		
SMS reminder messages with follow‐up or counselling	[35,36]	X	X		
EDM‐informed counselling	[37,38]	X	X		
Real‐time EDM with SMS reminders	[38–40]		X		
Electronic pharmacy refill tracking system	[41]		X		
Task shifting ART delivery	[29,42]			X	
Family‐level economic strengthening and savings program	[43]				X
Cash and non‐cash financial incentives	[44,45]				X
Nutrition education and/or food assistance	[46–48]	X			X
Agricultural and microfinance intervention	[49]	X			X

*This article is not a systematic review; some evidence‐based interventions may not be shown in this table.

SMS = short message service, EDM = electronic dosing monitor.

## Categories of adherence interventions

### Education and counselling

#### Overview

Education and counselling refer to communication strategies that may target ART initiation, dose‐taking execution, and/or persistence. *Education* is an exchange of information about medication to increase health literacy and reduce dosing errors. Although the efficacy of education‐only strategies is unclear [50,51], patient‐centered education (e.g. tailoring information to address concerns, use of relevant metaphors) is essential for standard medical treatment [52,53].

*Counselling* focuses on ART adherence‐related beliefs, attitudes, feelings, and skills in a collaborative patient‐counselor exchange. It involves (a) assessing needs and context, (b) information about behavior change, (c) facilitating adherence behavior change to the extent that the patient is able, willing and motivated, (d) identifying and modifying goals, and (e) arranging for ongoing assistance or changes in medication plans [54].

While adherence counselling is commonly included as a core component of ART delivery, provision of high‐quality counselling is challenging when the number of patients overwhelms providers, as is common in high burden RLS. Moreover, counselling is influenced by the counsellor's training and is difficult to standardize (e.g. style of delivery, tailored content). An immediate opportunity exists to improve medication adherence by augmenting, supporting, monitoring, and optimizing adherence counselling.

#### Promising and effective counselling approaches

##### Individual counselling delivered by trained interventionist(s)

A common model underlying individual counselling interventions is cognitive behavioral therapy (CBT) [55]. CBT uses a collaborative process in which a provider acts as coach and educator in helping patients identify thoughts, feelings, and behavior cycles that influence adherence [56,57]. Although more common in upper‐ and middle‐income countries, the use of CBT to promote ART adherence is growing in RLS with promising impact on adherence [20,22,58,59]. While expertise to train and supervise CBT counsellors is limited, software and computer‐assisted approaches may expand capacity (discussed below).

Motivational interviewing (MI) is another approach that has been used across diseases [60] and settings [61–63]. MI is a patient‐centered counselling style for eliciting behavior change by helping patients explore and resolve potential discrepancies between intentions and actual actions, as well as increase motivation to change behaviors that are inconsistent with those intentions; it is focused and goal directed, but not simply advice‐giving. MI may be brief (even a single session) or may be used in combination with other models, like CBT [64–66]. It can be delivered by workshop‐trained clinicians and does not require highly‐trained therapists [62]; however, optimal implementation requires ongoing support, which has been shown to be feasible in South Africa [64,67]. Adoption can be facilitated through publicly available and culturally adaptable training manuals and supervision tools [68], and is consistent with the skilled‐helper model already used in some LMIC. Evidence for the impact of MI on ART adherence, CD4 count, and/or viral suppression in developed settings is largely positive [69–74], though not completely [75]. Fewer studies have been undertaken in RLS, but positive effects on adherence were reported in Nigeria [23].

#### Peer‐delivered and family‐centered adherence counselling strategies

The WHO promotes peer support strategies for ART adherence among PLWH, particularly among key populations [18]. In RLS, individual peer‐to‐peer counselling and peer group support are among the most widely‐implemented adherence strategies [21,24,27–30,76–84]. Peer‐to‐peer support often includes counselling, psychosocial support (e.g. against stigma), and assistance with navigation of care, as well as community ART delivery, directly‐observed therapy, and defaulter tracking (some of which are discussed below). Titles for such providers vary and include community or peer health workers; treatment partners, supporters, or buddies; expert patients; and, in prevention of mother‐to‐child transmission (PMTCT) programmes, Mentor Mothers. Improvements have been seen in adherence, while effects on viral suppression have varied [21,24,27–30] and one intervention had no impact on adherence [81]. Adherence clubs, which often include peer‐to‐peer counseling, also tend to demonstrate high levels of acceptability and promise for promoting adherence and viral suppression [26,85,86]. More evidence is needed for assessing the impact of standardized peer counselling strategies on adherence in different PLWH populations in RLS, such as among youth [76,82] and pregnant/post‐partum women [87,88]. Furthermore, potential “turf‐wars” with professional healthcare workers about the scope of practice and competing roles [89] may need to be minimized through clear definition of roles and integration of peer counsellors into HIV programmes.

Family‐centered counselling may be particularly important for children and adolescents, as well as adults with concurrent challenges, such as mental illness [90,91]. One promising intervention delivered by lay staff has been developed in South Africa [92]. Another family communication intervention, originally developed to enhance coping with caregiver depression, has been adapted to improve child mental health and enhance family coping with HIV in Rwanda [93].

#### Looking to the future

Although not currently feasible in most RLS, optimized delivery of high‐quality adherence counselling with software or other technology‐enhanced strategies has recently received increased attention [94]. Laptop or tablet‐based counselling tools can facilitate counsellor‐patient interactions, provide engaging and learning‐based activities, and offer visual graphics for concept demonstrations. Preliminary data from one multimedia‐based, lay counsellor‐delivered intervention in South Africa showed improved pill count adherence [31,92].

Scalability of specific education and counselling strategies vary considerably based on current standard of care conditions. In many regions, cadres of para‐professionals, peers, and community support systems may be readily identifiable or already engaged in delivery of HIV care. Counsellors and nurse‐counsellors are increasingly common in care settings that serve large patient groups. As such, the education and counselling interventions identified often leverage existing resources. Optimizing delivery of these strategies by ensuring quality training and supervision, however, may require shifting available resources.

### Ict‐enhanced solutions

#### Overview

ICT‐enhanced solutions to support ART adherence include cellular phones for direct communication with individuals taking ART (e.g. automated short message services [SMS] and voice calls); electronic dosing monitors (EDM), which record the date and time of each monitor opening as a proxy for medication ingestion; and electronic pharmacy refill tracking systems – all of which potentially enable intervention delivery. EDM data can be retrieved periodically from some devices through a cabled connection to a computer or tablet, or in real time in other devices through a cellular connection. Given the wide availability of cellular networks globally and user‐friendliness of many ICT‐enhanced solutions, such approaches are promising as adherence‐promoting strategies [95,96] and have potential to improve all stages of adherence. For example, ART initiation may be promoted through SMS for linkage to care and monitored through EDM or pharmacy refill tracking systems, while SMS reminders/communication with or without monitoring can support dose‐taking execution and persistence over time [12]. Challenges to deployment include the need to keep cellular phones and/or EDM charged, gaps in reliable network coverage, and low education and literacy levels [97]. These challenges have been partially mitigated by community charging stations, phone sharing, and expanding cellular network availability. Misclassification of dose‐taking behavior may occur (e.g. taking multiple pills out of an EDM for later dosing, device non‐use, or inaccurately reported adherence via SMS). However, the detailed dosing histories obtained through EDM generally allow for objective, precise, and accurate identification of incomplete adherence [98,99].

#### Evidence for SMS

SMS communication to patients in RLS has demonstrated promise in improving dose‐taking execution [100–102], although the evidence is mixed. In Kenya, an intervention that used SMS to connect providers with PLWH and assess self‐reported adherence and health concerns showed increases in self‐reported adherence and viral suppression [35]. Another study of SMS reminders in Kenya showed improvement in adherence as assessed by EDM (used as a measurement tool) when the SMS reminders were sent weekly, but found no impact with daily reminders [33]. In Guatemala, daily SMS reminders improved viral suppression [32] and in Nigeria twice‐weekly SMS reminders increased self‐reported adherence [36]. However, other well‐conducted studies in Cameroon and India showed no adherence or virological benefit from scheduled reminders [103,104].

#### Evidence for EDM combined with counselling and/or SMS

Patients’ awareness of their adherence patterns can change their dosing behavior. A review and meta‐analysis of adherence enhancing interventions in 79 RCTs, in which electronically compiled drug‐dosing histories were assessed, showed that feedback to patients about their dosing patterns was the largest factor influencing adherence [105]. Several studies have confirmed that using EDM data to inform counselling improves ART adherence and CD4 cell counts [37,38,106].

Recent studies using real‐time EDM to deliver SMS triggered by late dose‐taking, however, have shown generally positive results. One study in China combined triggered reminders with data‐informed counselling and found an increase in EDM adherence [38]. A similarly designed study of EDM‐triggered SMS reminders in South Africa (that did not include supportive counselling) observed a decrease in treatment interruptions of >72 h, but no effect on overall adherence [40]. A pilot RCT in Uganda that tested both scheduled reminders and triggered reminders combined with real‐time EDM found improved adherence and a decrease in treatment interruptions only with scheduled reminders [39]. None of these EDM studies was powered to assess viral suppression. Additionally, a recent four‐arm study using a mix of EDM and SMS for individuals receiving tuberculosis treatment in China found a reduction in incomplete adherence using a combined EDM‐SMS approach, but no effect from SMS alone [107].

#### Understanding the mixed evidence

The variation in these studies may stem from the timing and/or content of SMS messages, existing adherence barriers in each population, and other individual or cultural preferences for the SMS or monitoring [108]. In a review of global SMS interventions for adherence in conditions not restricted to HIV, two‐way communication (versus one‐way messaging) was found to be the main driver of SMS‐based adherence improvements [109]. Further assessments are needed with both scheduled and triggered SMS reminders/communication in different settings and populations, along with testing of combination approaches.

#### Electronic pharmacy refill tracking systems

Another promising technology is use of electronic pharmacy refill tracking systems. These systems have been used in Haiti to differentiate ART failure risk and could be linked to alerts for clinical follow‐up [41]. They have also been shown to accurately reflect retention in care in Malawi and South Africa [110,111], thereby reducing staff time and effort.

#### Looking to the future

Currently, approaches that rely on cellular phones and existing computer record systems are more affordable than EDM‐based platforms, and thus have greater potential for immediate scale up. Both EDM and electronic pharmacy refill tracking systems have the advantage of generating real‐time adherence information, and may identify patients for targeted use of viral load testing and/or prompt intervention *prior* to virologic failure. Over time, the ability to focus resources on patients at high risk of virologic failure, as well as the probable decline in technology cost, may make these interventions cost‐neutral or cost‐saving [112]. Importantly, recent modelling found that EDM would be cost‐effective with or without the availability of viral load testing at <US$50 [113]. Additionally, ICT‐enhanced solutions have generally been acceptable to PLWH [114], which will facilitate scale up, although privacy (e.g. unintended disclosure of HIV status resulting from EDM use) and other concerns may be an issue in some settings or populations [115,116]. Both EDM and SMS have generally been found to create a sense of “connectedness” to clinics [117,118] and to motivate patients who appreciate “being watched”. These findings – and the increasing connectedness of people through technological advances globally – suggest that electronic interventions, whether harnessing existing phones or putting devices in patient's hands, have potential for reaching and supporting large numbers of PLWH.

### Healthcare delivery restructuring

#### Overview

The organization of people, institutions, and resources that deliver healthcare, referred to broadly as healthcare delivery systems, can affect adherence [119–122]. For example, long wait times at clinics, fragile supply chains leading to stock outs, or extended travel to clinics can make it difficult to obtain care and avoid treatment interruptions. Patient interactions with healthcare workers can also influence adherence [123–125]. In many RLS, healthcare delivery systems remain poorly equipped to provide long‐term chronic disease care, including HIV care many are overburdened by high patient loads and limited resources [126]. This burden has increased with rapid ART scale‐up, more inclusive ART eligibility, and plateaus in international funding [127].

Improvements in structural, logistical, and interpersonal elements of healthcare delivery can improve the overall efficiency of crowded service delivery centers. For example, decentralized services decrease the demands on patients for care seeking, such as transportation and time away from work [128]. Other techniques, such as point‐of‐care CD4 count testing [129] and integration of related services (e.g. antenatal care and PMTCT programmes; tuberculosis and HIV treatment programmes [130,131]), improve linkage to and retention in care. Likewise, emerging interventions such as differentiated models of care (discussed below) are designed to improve efficacy of treatment programs. While not necessarily targeting adherence, these interventions enable better care delivery.

#### Evidence for current adherence interventions

Several intervention studies and pilot programmes have shown that streamlining and reducing the burden of clinic visits and medication refills are well‐received and specifically effective at promoting adherence. Most involve community‐based or alternative care delivery models and task shifting to nurses [29,42,121]. In some delivery models, PWLH with perceived good adherence (e.g., those who maintain viral suppression) can obtain ART via an adherence club [26,85,86]. These clubs enable a “fast‐track” refill mechanism, as well as provide adherence counselling and peer support, as mentioned above. They have been found to reduce losses to follow‐up and virologic rebound in already virologically suppressed patients [26]. Adherence clubs, however, are unlikely to increase poor adherence levels without concurrent counselling. Similar fast‐track, nurse‐led care has been used successfully to reduce loss to follow‐up and death among high‐risk patients initiating ART with low CD4 counts [132]. Another approach shown to reduce the care burden without compromising care uses a group‐based shared responsibility model, in which one group member collects symptom reports and obtains ART refills for all group members [42]. In yet another model, patient‐defined support networks improved clinic attendance [28].

#### Looking to the future

Patient‐oriented adaptations of healthcare delivery systems can overcome structural barriers, reduce transportation costs and waiting times, and ultimately lessen treatment fatigue and losses to follow‐up. These adaptations can also increase time and/or resources for disclosure, treatment education and social support – all of which are beneficial for ART retention and adherence. Moreover, they may improve patient care if the efficiencies gained reduce the burden on providers and improve the provision of additional or improved services. Implementation science research that evaluates these adaptations at scale and assesses cost‐effectiveness is needed, as are partnerships with local Ministries of Health and other stakeholders who are key to systems‐wide changes [133]. Changes in healthcare delivery often require resources from multiple aspects of healthcare systems, thus limiting scalability. However, once changes have been established and produce desired outcomes, they can be self‐sustaining by freeing otherwise encumbered resources.

### Economic incentives and social protection interventions

#### Overview

Economics, psychology, and social inclusion theories guide the development and implementation of economic‐based interventions. A broad consensus is emerging to suggest the need to differentiate between the impairments inflicted by poverty [134] and the avenues to address poverty, which include conditional and unconditional incentives (i.e. cash transfers); food security and livelihood support; and social protection programmes [135,136]. Social protection is generally understood as public actions to address poverty, economic shocks, and social vulnerability [137]. Income or in‐kind support programmes directed at these concerns can help increase access to services and thereby increase ART adherence. Contingency management interventions use incentives to motivate behavior change to counter individual behavioral choice [138,139]. More broadly, conditional cash transfers at the national level are designed to reduce poverty, increase social protection, and improve education outcomes [140], thereby improving HIV treatment outcomes.

#### Unconditional and conditional economic incentives

Current incentive practices vary widely by type, amount, length of duration, and conditionality. Compared to conditional economic incentive programmes that use specific eligibility criteria, unconditional economic incentives may have lower administrative burden, thus favoring scalability, while producing similar results, as seen in rural Kenya and with adolescent girls in Malawi [141,142]. Non‐cash transfers (e.g. loans plus training, in‐kind loans, forgivable grants plus training) can also be used as incentives. Some of these models (in some cases including EDM) are being tested for impact on adherence‐related behaviors and other HIV‐related outcomes in RLS [43,143]. With any incentive program, it is important to recognize that incentives can motivate patients to misreport their circumstances to qualify for programmes. The problem of misreporting can be mitigated by using objective eligibility measures (e.g. biological testing) or other indicators verified by a third party [141], or by making the programmes universal (i.e. non‐means tested) [144].

In the United States, five RCTs of conditional economic incentives have demonstrated increased ART adherence [145], though one community‐based trial had mixed results [146]. Less is known about the application of cash incentives conditional on adherence in RLS. One RCT in India among substance users showed increased linkage to care and ART initiation, but no difference in viral suppression [45]. Preliminary results from an RCT among adults in Uganda, however, found improved adherence with small prize incentives [44]. In a large RCT of unconditional cash transfers versus a wait‐list control in Uganda, cash transfers had no effect on adherence, retention, or mortality, although the study found a small, significant decrease in CD4 cell rebound among transfer recipients [147]. Importantly, even if cash transfer programmes are effective in RLS, sustainability and scalability without a steady influx of resources from donors or governments is unclear, particularly where political will is lacking [136]. The use of small incentives or lotteries may keep costs low and increase potential for scalability.

#### Food security interventions

Food insecurity is a critical determinant of incomplete ART adherence in RLS [148–152]. Some economic interventions take the form of in‐kind loans or grants, like food vouchers and targeted food assistance, which have been recommended for HIV treatment packages in RLS [153–155]. Studies in sub‐Saharan Africa, Haiti, and Honduras have found that food supplementation delivered as part of HIV care can lead to better ART adherence and improved clinic attendance [46–48]. A study in Zambia showed that more participants in the food supplementation group achieved high adherence compared to controls [47]. In a summary of the evidence on food assistance and adherence to both HIV and TB therapy, eight of ten studies found that food provision improved adherence to ART or TB treatment completion [156].

Notably, while cash transfers and food supplementation may be appropriate during early linkage to care and ART initiation, they are limited in scalability and sustainability in the absence of ongoing government or donor funding commitments. Moreover, they do not address upstream determinants of food insecurity [148–152].

#### Promising approaches to economic and livelihood interventions

Livelihood interventions that include microloans and/or business skills training are a promising strategy to help families overcome economic barriers to sustained adherence. Given the political and financial challenges of conditional cash transfer interventions, agricultural interventions and savings‐led groups may have a better chance of sustainably improving health outcomes at scale.

While data are limited, one recent study of an agricultural and microfinance intervention in Kenya found significantly improved viral suppression, CD4 counts, and food security among PLWH [49]. In an ongoing longitudinal qualitative study, this intervention also improved ART adherence and clinic attendance, and reduced HIV‐related stigma and discrimination [157]; its impacts on stigma were hypothesized to occur through restoration of health, increased social integration, and an increased ability to make reciprocal economic contributions to community safety nets [158]. The same study also found changed gendered power dynamics, reduced high‐risk behaviors, and improved prioritization of health [157,159].

The specifics of which poverty‐alleviation interventions are scalable and most effective are being evaluated in clinical trials (e.g. NCT01957917; NCT01790373) and may vary by country and context. Identifying and addressing economic barriers to health and evaluating the impact of different incentive and livelihood‐promoting strategies on medication adherence are key research needs. Ultimately, interventions implemented at the health systems level may make the most difference at scale. For example, the national conditional cash transfer programme in Mexico, Oportunidades, in operation since 1998 and covering over 25 million people, is associated with higher affiliation to Seguro Popular (the Universal Health Insurance Program for the Poor) [160]. That programme, in turn, has enabled Mexico to provide universal ART access [161]. Similarly, national level ART investments since 1996 have provided population benefits in Brazil [162].

## Targeted and tailored adherence support

Some adherence support may be useful for all individuals taking ART; however, most interventions should be focused on those who struggle with adherence, thus enabling more directed and cost‐effective implementation. Interventions should also be combined and tailored when possible to optimize close matching between the preferences and capacities of PLWH, providers, and healthcare delivery systems. Treatment initiation, dose‐taking execution, and treatment persistence are different behavioral processes and may best be served by different approaches. Many individuals fail to persist with treatment relatively soon after treatment initiation [163–165]. This phenomenon suggests that initiation is a particularly vulnerable time during which extra efforts to keep PLWH engaged with their care are warranted. For example, assistance with integrating medication taking into every‐day life, learning to cope with potential HIV‐related stigma, and dealing with potential side effects may be sources of early non‐persistence. PLWH who remain on treatment, but with gaps, may have different problems, such as difficulty finding transportation to refill medications, a perception that improved health renders regular medication taking unnecessary, or long‐term side effects, such as lipodystrophy. Individuals who interrupt therapy may quit care altogether, necessitating efforts to find and re‐engage them. Moreover, needs and preferences of healthcare systems and cultures may differ. For instance, the strength of existing counselling programmes varies across institutions. Some groups may respond better to messages delivered through community‐based theatre or music, while others prefer individual counselling. Technology acceptance may be high in one area and low in another. Understanding these varying preferences is critical for choosing adherence interventions.

Appropriately targeted and tailored adherence support requires accurate measurement of adherence. While many adherence measurement tools exist, none is perfect [166]. The strengths and limitations of each measure are listed in Table 2. Importantly, “average” or “good” adherence (e.g. 80%) can be misleading, particularly when based on self‐report, pill counts, and other methods with potential for strong positive bias. Adherence patterns (i.e. sustained interruptions), including treatment discontinuation, are generally more important than average adherence in determining risk for viral rebound [7,167]. Additionally, measures obtained during clinical care do not reflect challenges for those lost to follow‐up, and thus likely represent underestimates.

**Table 2:** Strengths, limitations, potential advances, and other considerations for adherence monitoring tools that may be used for targeted and tailored support.

Monitoring tool	Strengths	Limitations	Potential advances/considerations
*Subjective*			
Self‐report	Easy and relatively inexpensive to collect Reported missed doses are likely accurate [169] Correlates with viral suppression in some contexts [170–173]	Generally overestimates adherence due to social desirability and recall bias [174,175] Difficult to assess patterns of adherence Collected too infrequently to reliably detect incomplete adherence, non‐persistence, or risk of virologic failure [176]	SMS may decrease social desirability and recall biases, as has been shown with other forms of technology [177,178]
*Objective*			
Pill counts	Easy and relatively inexpensive to collect Returned pills strongly suggest incomplete adherence	Tends to overestimate adherence due to social desirability bias, i.e. “pill dumping” [179,180] Provides only an average adherence and cannot assess patterns	Unannounced pill counts are less subject to manipulation, but are resource intensive [181]
Pharmacy refill	Already collected by most clinics Reveals failures of ART initiation and persistence Correlates with clinical outcomes [182] Useful for population trends and specific poor performing groups (e.g. a WHO “early warning indicator”) [183]	Provides maximal predicted average adherence and may miss incomplete adherence Many existing pharmacy systems are not optimized for tracking	Could be made actionable for intervention deployment if made available to clinicians or community health workers
Electronic dose monitoring	Only method to provide day‐to‐day patterns, which better predict the risk of virologic failure compared to the average adherence [7,167,184,185] Allows for tailored counselling and intervention deployment, potentially in real time	Currently expensive and resource‐intensive Technical challenges (e.g. battery failures) and device non‐use (e.g. “pocket dosing”) may limit accuracy	Additional development needed to improve technology and reduce cost for use in clinical settings Could be used intermittently to reduce costs (e.g. during ART initiation or only for intensive support)
Drug detection	Only measure to directly assess drug ingestion (e.g. plasma, hair, dried blood spots) [186,187]	Currently expensive and resource‐intensive Some methods reflect recent (e.g. plasma indicates ~3 half‐lives) rather than typical dosing Provides only average adherence Inter‐ and intra‐individual variability limit interpretation [188] Information generally not available until after the patient encounter, making interventions challenging	Additional research needed for use in clinical settings, including point‐of‐care methods and regional laboratory capacity
Viral load^a^	Indicates sufficient adherence for clinical benefits for guiding targeted and tailored support [189]	Currently expensive and resource intensive, especially over a lifetime [190] Information generally not available until after the patient encounter, making interventions challenging Incomplete adherence may not be detected until after drug resistance has developed, leading to a need for expensive, often unavailable second‐line ART Lack of drug resistance testing limits interpretation	Low‐cost, point‐of‐care viral load monitoring would increase access, allowing for complementary role along with adherence monitoring and interventions

^a^Viral load is not a direct measure of adherence. Rather, it indicates sufficient adherence to achieve viral suppression, which signals success in achieving the clinical benefits of ART.

**Comments are drawn from the literature as cited, as well as overall reviews [** **8**,**166**,**168** **]**

## Implementation and evaluation of adherence interventions

**Figure 1:**
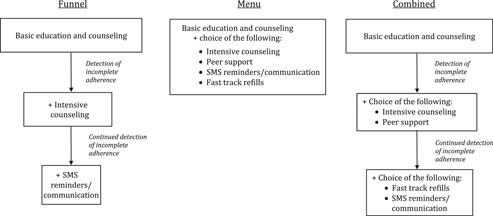
Examples of funnel and menu approaches for targeted and tailored implementation of ART adherence interventions (other interventions could be substituted). With a *funnel approach*, all individuals would receive basic education and counselling. Based on one or more adherence measures (see Table 2), individuals identified with incomplete adherence would receive a standard intervention. The intensity of intervention would then increase for the likely decreasing number of individuals who continue to have adherence challenges. With the *menu approach*, a number of intervention options could be presented to individuals, allowing them to choose their intervention strategy. Menus could be tailored for specific populations (e.g., individuals with depression may opt for counselling, those with structural barriers may benefit from adherence clubs). A *combined approach* with basic and more intensive strategies offered as a menu at each level of the funnel could be considered.

Figure 1 provides schematics of two general approaches to consider in choosing adherence interventions for optimally targeted and tailored support: the funnel and the menu. With a funnel approach, all individuals receive basic ART education and counselling and are screened for incomplete adherence by one of the above‐noted monitoring tools. Those identified with incomplete adherence (which may include those not initiating care) receive a standard intervention. For those continuing to experience adherence challenges, subsequent interventions of increasing intensity may be needed. Of note, the first intervention should be appealing to dissuade PLWH from missing medication to gain benefits of later steps in the funnel (e.g. incentives). With the menu approach, a number of options are offered, allowing individuals to choose their intervention strategy. Menus could be tailored for specific populations. Combined approaches with basic and more intensive strategies available as a “menu of options” at each level of the funnel might also be possible. Strategies are adapted to local cultures and communities, addressing setting‐specific challenges and leveraging local resources. A process of continuous quality improvement is advisable to help optimize the strategies for a given population or setting, such as a Plan‐Do‐Study‐Act cycle [191,192].

Several techniques will assist in the evaluation of adherence intervention implementation. First, adaptive and sequential research designs within implementation science frameworks may be particularly efficient and informative; such trials are ongoing (e.g. NCT01904994, NCT02338739). Second, study designs typically focus on the short term (i.e. one year or less) and miss important challenges, as well as long‐term positive effects; longer follow‐up is critical for understanding more comprehensive impact. Third, research results often fail to translate into real‐world changes because they are typically conducted in relatively well‐resourced settings and with relatively high‐functioning patients; future studies need to be conducted in generalizable settings [193]. Finally, all interventions should be assessed with clearly defined adherence and adherence‐related outcomes (e.g. reduction of unmet needs, positive attitudes and beliefs about care, cost‐effectiveness) in addition to viral suppression.

## Consideration of specific populations

ART adherence interventions have thus far been presented with little reference to specific populations (which is beyond the scope of this article). Adherence monitoring and interventions may require adaptation for different populations and research in these areas often lags that in the general population. In particular, few evidence‐based interventions have been developed to promote adherence in children and adolescents [76,194], for whom interventions shown to be effective in adult populations may not be helpful [195]. While increased adherence monitoring and support may be disproportionately needed for key populations, not every member of a key population needs help. Tremendous variation exists within vulnerable populations, and among those believed to be at low risk, especially as life circumstances change over time. Additionally, implementing interventions at scale for some populations may prove quite challenging, thus requiring more resources.

## Discussion/conclusions

The evidence base is promising for currently available, effective, and scalable ART adherence interventions for RLS. Participants at the meeting identified numerous interventions that build on existing healthcare infrastructure and leverage available resources. Those most widely studied and implemented to date involve peer counselling, adherence clubs, and SMS. Many additional interventions could have an important impact on ART adherence with further development, including standardized counselling through multi‐media technology, electronic dose or pharmacy monitoring, decentralized and differentiated models of care, and livelihood interventions. Although cost can pose a barrier to implementing adherence interventions, the value of supporting high adherence and associated viral suppression warrants consideration of investment, particularly as the technology to support these efforts becomes more affordable. Scalability of these interventions will need to be assessed outside of the research context and in settings with limited additional resources [193]. Even when immediate resources do not include critical intervention ingredients (e.g. peers, adherence clubs), retooling, shifting, and reallocation of resources can identify avenues for sustainable new or improved intervention approaches. When combined with differential care delivery strategies, new resources can be potentially discovered.

A key area for development is more active and accurate adherence monitoring to enable the targeting of adherence support for those with the greatest need. Real‐time EDM and electronic pharmacy refill tracking systems are two ways to achieve such precise monitoring [196]. Because only a minority of patients miss sufficient doses of ART to threaten viral failure, selective targeting of interventions based on adherence assessments allows for greater investment in more resource‐intensive interventions for those patients who truly need them, with the potential for differential, patient‐specific, and cost‐saving interventions. Engagement in care is critical, but engagement alone does not ensure the adherence required for sustained viral suppression. Moreover, adherence may change over time and is subject to disruptions in routine (e.g. sentinel life events, like a death in the family). Newer antiretroviral medications (e.g. integrase inhibitors) may be available in RLS in the near future, allowing more forgiveness for missed doses [197]; however, feasible and acceptable monitoring and associated support strategies will still be needed over a lifetime of treatment.

Implementation of adherence interventions at scale will face important challenges. First, healthcare delivery systems, at both regional and national levels, will have to be receptive and committed to widespread implementation. Human and financial investment must be weighed carefully, making cost‐effectiveness and the overall impact of candidate interventions critical. Second, monitoring and evaluation of ongoing adherence programmes, as well as research into new interventions, will require funding beyond what is typically invested in single intervention studies. One of the goals of this article is to emphasize both the need for this investment, as well as its potential benefits.

Given the availability of encouraging and robust evidence, the opportunity exists today – more than ever before – to address and resolve many of the challenges to effective ART adherence, especially as access extends to increasing numbers of PLWH. We now have an opportunity to draw on a wealth of tested ART adherence technologies and interventions to expand support to PLWH so that adherence challenges do not limit the potential of ART to help bring about the end of AIDS.

## Competing interests

Author BV reports working for WestRock and author BT reports working for the Arcady Group – both of which manufacture electronic dose monitors. No other authors report competing interests.

## In memoriam

Professor John Uruhart, M.D., Dr. h.c.(Utrecht), FRCPE, a pioneer in the field of adherence passed away on March 19, 2016. His seminal publications on taxonomy, forgiveness and measurement‐guided patient management, along with the introduction of MEMS® electronic dose monitors in 1988, have had and will have a long‐term impact on this field.

## Acknowledgements

The authors would like to thank Heather Thompson for organizing the meeting and Jenna Sherry, Bridget Burns, Radka Stepanska, and Lindsey Garrison for facilitating this article. Peter Ehrenkranz and Dan Hartman provided critical review.

The meeting on which this paper is based was funded by the Bill and Melinda Gates Foundation.
